# The hidden malaria: misidentification of *Plasmodium malariae* and *Plasmodium ovale* in low-transmission setting targeted for elimination in Ethiopia

**DOI:** 10.1186/s13071-026-07511-0

**Published:** 2026-08-03

**Authors:** Hallelujah Getachew, Chloe Wang, Kassahun Habtamu, Assalif Demissew, Teshome Degefa, Solomon K. Birhanie, James W. Kazura, Christopher L. King, Guiyun Yan, Delenasaw Yewhalaw

**Affiliations:** 1 School of Medical Laboratory Sciences, Institute of Health, Jimma University, Jimma, Ethiopia; 2 Department of Medical Laboratory Sciences, Arba Minch College of Health Sciences, Arba Minch, Ethiopia; 3 Tropical and Infectious Diseases Research Center (TIDRC), Jimma University, Jimma, Ethiopia; 4 Department of Population Health and Disease Prevention, University of California at Irvine, Irvine, CA 92697 USA; 5 Menelik II Medical & Health Science College, Addis Ababa, Ethiopia; 6 Department of Medical Laboratory Sciences, College of Medicine and Health Sciences, Ambo University, Ambo, Ethiopia; 7 West Valley Mosquito and Vector Control Agency, Ontario, CA USA; 8 Center for Global Health and Diseases, Case Western Reserve University, Cleveland, OH USA; 9 Veterans Affairs Medical Center, Cleveland, OH USA

**Keywords:** *Plasmodium malariae*, *Plasmodium ovale*, Species misidentification, qPCR, Ethiopia

## Abstract

**Background:**

Quartan malaria caused by *Plasmodium malariae* (Pm) and tertian *Plasmodium ovale* (Po) malaria has been largely overlooked in Ethiopia due to their milder clinical symptoms, lower prevalence, and frequently misdiagnosis during routine microscopic. This study estimated the prevalence of Pm and Po infections misdiagnosed as *P. vivax* (Pv) in selected health facilities in southwestern Ethiopia.

**Methods:**

A cross-sectional study was conducted among study participants who were positive for Pv by microscopy or rapid diagnostic test (RDT) and who were attending health facilities within Arjo-Didessa sugarcane plantations and surrounding areas in Oromia, southwestern Ethiopia, between September 2019 and July 2022. Of the 3590 febrile patients attending health facilities, 323 were Pv positive by microscopy or RDT. Among these, 245 Pv-positive samples were re-evaluated using quantitative polymerase chain reaction (qPCR). Species-specific primers targeting Pv, *P. falciparum* (Pf), Pm, and Po were used for species confirmation.

**Results:**

From 245 vivax malaria cases, RDT were performed to 162 sub-samples, among them 8.6% (14/162) were negative by RDT. None of the samples tested by RDT and microscopy detected Pm or Po. Of these 245 tested and reported as Pv positive by microscopy, qPCR confirmed that 215 (87.7%, 95% CI 82.98–91.58) were positive for Pv, 9 (3.7%, 95% CI 1.69–6.86) for Pf, 8 (3.3%, 95% CI 1.42–6.33) for Pm, 6 (2.4%, 95% CI 0.90–5.25) were positive for Po, and 7 (2.8%, 95% CI 1.16–5.80) had mixed infections (Pf and Pv).

**Conclusions:**

The findings of this study highlights the need for a strategic shift to integrate Pm and Po into Ethiopia's malaria control and elimination program. Moreover, strengthening the diagnostic capacity should include training of microscopists for accurate *Plasmodium* species identification and adopting highly sensitive diagnostic tool for all four *Plasmodium* species in resource-limited settings. This is essential for enhancing malaria control and accelerating progress toward elimination.

**Graphical Abstract:**

**Figure:**
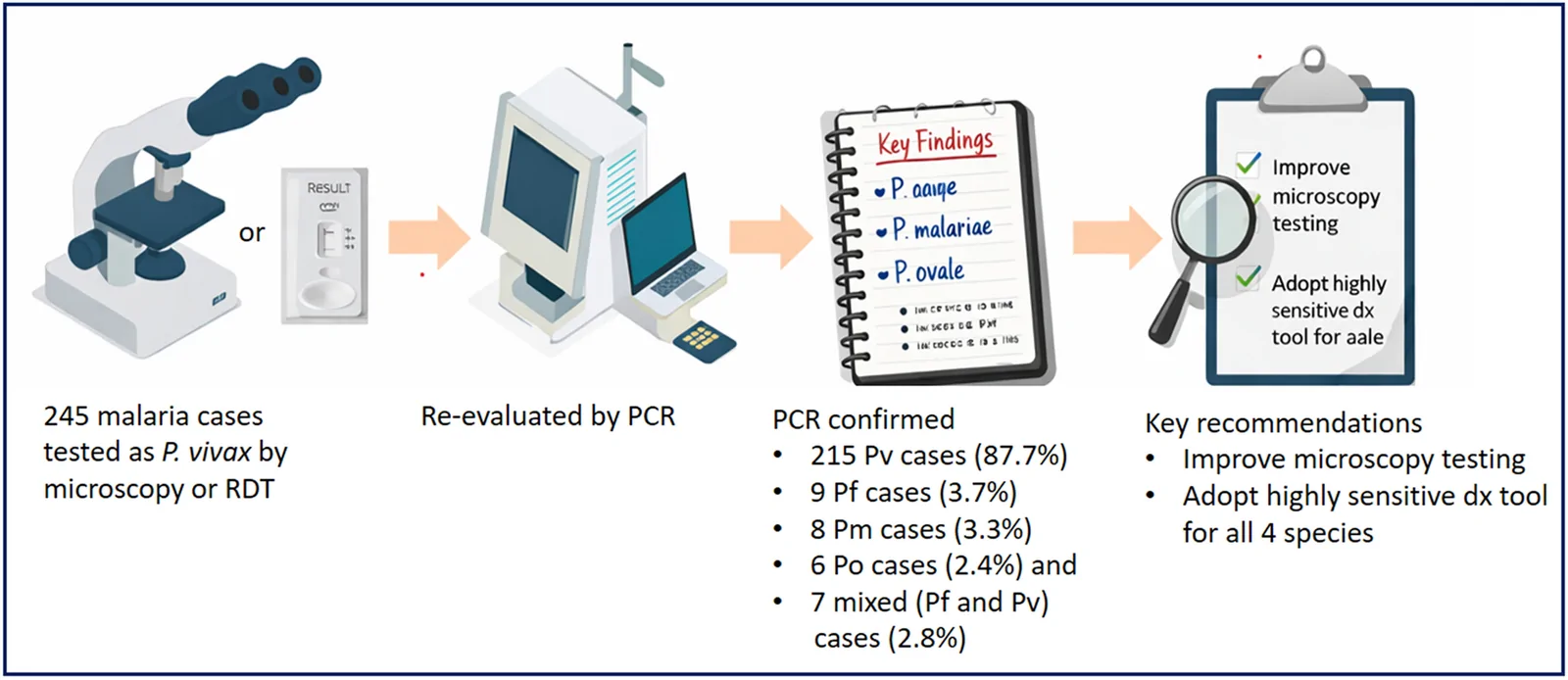

**Supplementary Information:**

The online version contains supplementary material available at https://doi.org/10.1186/s13071-026-07511-0.

## Background

Malaria control and elimination strategy mainly focuses on *Plasmodium falciparum* (Pf) and to a lesser extent on *Plasmodium vivax* (Pv) [1]. *Plasmodium malariae* (Pm) and *Plasmodium ovale* (Po) are largely overlooked due to their milder clinical symptoms, lower prevalence, and are frequently misdiagnosis during routine microscopic examination. Pm and Po are endemic across tropical and subtropical regions, coexisting with other *Plasmodium* species [2]. The control of Pm and Po is challenging due to unavailability of Pm or Po species-specific routine diagnostic methods, low-grade parasitemia, and a potential asymptomatic nature of the infection [3–6].

Pm clinical manifestation can vary widely, from asymptomatic to severe form of infection. The severe manifestation associated with nephrotic syndromes in young children and adults [7–11] and more long-lasting and chronic illness associated with anemia and even mortality [12, 13]. A case report of severe manifestation of Po was also documented from central India and Malaysia [14, 15]. In recent years, the incidence of Pm and Po has increased across the sub-Saharan Africa. This shows the invasion of these parasites could be Duffy antigen independent pathways [16, 17]. Molecular surveys have confirmed co-circulating Pm and Po*,* suggesting the rising prevalence in some regions where Pf has declined [1, 4, 6, 18, 19]. For instance, unusually high proportions of Pm clinical cases have been reported in Colombian Amazon Region (43.8%; 294/675) [20] and in Vietnam (46%; 164/356) [21]. This suggests that the transmission patterns of the non-falciparum species do not necessarily follow those of Pf, stressing the need for attention toward Pm and Po malaria [1].

In Ethiopia, the two predominant *Plasmodium* species are Pf and Pv with a general proportion of 60–70% and 30–40%, respectively [22]. Pm and Po are considered to be present at a very low prevalence with focal and localized distribution, and often overlooked. The first report on the presence of Pm in Ethiopia was documented in 1938 in southern Ethiopia [23], and then later reported in the late 1960s in western and northwestern areas of the country [24, 25]. On the other hand, the presence of Po was reported from south and northwestern Ethiopia in the late 1960s [24]. More recently molecular studies indicated the presence of both Pm [26] and the two genetically distinct forms of Po in the country [27, 28]. Therefore, the focus of malaria diagnosis, treatment, and control strategy need to consider the importance of Pm and Po in the country especially in areas targeted for malaria elimination. Malaria elimination requires the disruption of onward transmission by detecting every infection and proper case management [29]. Accurate species identification and understanding of parasite species distribution are thus important to ensure efficacy of malaria elimination efforts.

In Ethiopia, malaria diagnostic approach varies across levels of the health care system. Health centers and hospitals perform diagnosis by microscopy, while health post uses rapid diagnostic test (RDT) for the diagnosis of Pf and Pv. At the national and regional referral level, molecular tests are performed [30]. Although RDT require minimal training, no electricity, and are rapid compared to microscopy [31], RDT has limitation on the performance for the detection of Pm and Po malaria [32–34] and does not quantify parasite. In resource-limited setting microscopy remains the golden standard for malaria diagnosis. An expert microscopist can detect up to 5–10 parasites per μl of blood, while an average microscopist may detect > 100 parasites per μl [35]. However, microscopic diagnosis has often been challenged by the misidentification of Pm and Po because it requires continuous training to ensure proper diagnosis. On the other hand, malaria RDT is reported to detect > 100 parasites per μl, whereas molecular tests such as qPCR can detect < 5 parasites per μl [35]. It is often not feasible to use the highly accurate and precise technique such as qPCR at the primary health care system because it requires a special training, advanced laboratories and resource intensive [17, 36]. Therefore, the aim of this study was to estimate the prevalence of Pm and Po infections misdiagnosed as Pv in selected health facilities in southwestern Ethiopia.

## Methods

### Study area and design

A health facility-based study was conducted targeting febrile patients attending seven selected health facilities around Arjo-Didessa sugarcane irrigated area and the surrounding localities in southwestern Ethiopia (8°36′0’’ N, 36°24′0’’ E). The Arjo-Didessa sugar plantation covers more than 4000 hectares of land, with a planned future expansion of 80,000 hectares. Health facilities such as Arjo-Didessa Sugar Factory Clinic, Command 2 Health Post and Command 5 Health Post, were found within the irrigation project and gave service to sugar plantation workers. Most of the sugar plantation workers were migrant workers, who stayed in the area for 1–6 months. These migrant workers were seasonal and casual workers. The seasonal workers come to the area during the planting and harvesting season and work for up to 3 months under a contract. Casual workers work on a weekly basis. However, health facilities such as Abote-Didessa Health Post, Hunde-Gudina Health Post, Kerka Health Post, and Sefera-Tabiya Health Post give service to the surrounding local communities. The study was carried out between September 2019 and July 2022. A detailed description of the study site has been published elsewhere [37]. Malaria transmission in the study area is largely seasonal, low, and unstable, with peaks from September to December and minor surge from April to June [38].

### Study participants

Study participants were recruited from the routine health facilities visits: health posts and health center. A total of 3.590 self-presenting febrile patients attending Arjo-Didessa Sugar Factory Clinic, Command 2 Health Post, Command 5 Health Post, Abote-Didessa Health Post, Hunde-Gudina Health Post, Kerka Health Post, and Sefera-Tabiya Health Post were screened for malaria during the study period. Patients above the age of 3 months and attending the selected health facilities from September 2019 to July 2022 were eligible for the study. Any patient reported as being positive for Pv using microscopy or RDT was asked for voluntary participation in the study. During enrollment, trained data collectors administered a structured questionnaire to the consenting Pv mono-infected participants to obtain sociodemographic information and data on malaria control measures. Then capillary blood samples were collected for microscopy and qPCR analysis.

### Sampling

A total of 245 capillary blood samples were collected on filter papers from microscopy or RDT confirmed vivax malaria outpatients for molecular diagnosis during the study period. The collected capillary blood sample on filter papers were air-dried, placed in separated plastic bags containing silica gel, and stored at −80 °C for further analysis.

### Rapid diagnostic test (RDT)

Capillary blood sample was collected by finger prick and 5 μl blood was added into the sample well of multi-species CareStart^™^ Malaria Pf/Pv (HRP2/pLDH) Ag Combo RDT test kits (Access Bio Ethiopia, INC.) that detect PfHRP2 and PvLDH. Then two drops (60 μl) of buffer were added into the buffer well. Finally, the test result was read in 20 min. Interpretation of the results were done according to the manufacturer instructions.

### Microscopic examination

Thick and thin blood films were prepared and stained with 10% Giemsa solution and a medical laboratory technologist examined under light microscope at Arjo-Didessa Sugar Factory Clinic. The normal protocol for microscopic diagnosis at the health facilities was followed. Parasite density was not evaluated because the routine work of health facilities in rural areas of Ethiopia involves checking for the presence or absence of parasites but does not extend to calculating parasite densities. A second highly trained medical laboratory technologist from Jimma University rechecked all positive slides.

### DNA extraction

For the DNA extraction procedure, modified Chelex-100 resin was utilized [39]. Using a puncher, one dot of the dried blood spot from filter paper was cut into piece that were roughly 3–5 mm in size. After each sample, the puncher was cleaned by punching paper ten times. After transferring the blotted filter paper to a 1.5 ml Eppendorf tube, 50 µl of 10% saponin and 950 µl of phosphate buffered saline (PBS) were added to lyse the red blood cells. The sample was mixed and then incubated for four hours or overnight at 4 °C. After centrifuging the mixture for 10 min at room temperature at 14,000 rpm, the supernatant was removed. After adding 1 ml of PBS and centrifuging at 14,000 rpm for 5 min, any remaining saponin was discarded. By centrifuging for roughly 15 s, the leftover PBS was discarded. The filter paper was allowed to dry at room temperature for 15 min. To extract the parasite DNA, 150 µl of 20% Chelex suspension and 100 µl of distilled water was added. The mixture was then incubated at 95 °C in a water bath for 10 min, and vortexed it every two minutes. After centrifuging the mixture for one minute at 14,000 rpm, 200 µl of the supernatant (DNA) was transferred to a 0.5 ml tube and kept at −20 °C for qPCR analysis.

### Multiplex qPCR amplification

*Plasmodium* species were identified using multiplex qPCR. Each DNA sample's was amplified using primers based on 18S rRNA genes. Two rounds of amplification were carried out. Round one was used to identify Pv and Po. A second round was to identify Pv and Pm. In round one, a total of 12 μl qPCR reaction mixture containing 2.1 μl genomic DNA, 6 μl FastAdvanced MM (2X), 0.4 μl forward and reverse primers for Pf, Pv, and Po, each (Var-F, Var-R, Pv-1, Pv-2, Po-1, and Po-2), and 0.5 μl of each probe (Pf_varAST_Probe, Pv-Probe, and Po-probe) for Pf, Pv, and Po*,* respectively, was used [40, 41]. In the second round, a total of 12 μl qPCR reaction mixture containing 2.1 μl genomic DNA, 6 μl FastAdvanced MM (2X), 0.4 μl forward and reverse primers for Pf, Pv, and Pm each (Pf-1, Pf-2, Pv-1, Pv-2, Pm-1, and Pm-2), then 0.5 μl of each probes (Pf18S_Probe, Pv-Probe, and Pm-probe) for Pf, Pv, and Pm was used. Quant Studio 3, Applied Biosystems Real-Time PCR, the reaction profile were set as follows: 50 °C for 2 min, 95 °C for 2 min, 95 °C for 3 s, and 60 °C for 30 s followed by 45 cycles for each round 1 and round 2 amplification process. Sequences of primers are shown in Supplementary Table S1.

### Data processing and analysis

Data were checked for completeness and consistency before analysis; then all the data from study participants were imported into Excel. The statistical analysis was performed by STATA (version 17) and GraphPad Prism (version 9.5.1). Descriptive statistics were used to assess the distribution of the sociodemographic characteristics and independent variables as well as proportion of malaria-positive samples with 95% confidence intervals (CIs).

## Results

Of the 3,590 febrile patients attending health facilities, 323 were Pv positive by microscopy or RDT out of which 245 Pv cases were re-evaluated by qPCR. Among the 245 cases enrolled in this study, the median age of the study participants was 20 (interquartile range (IQR) 13.5–28). The majority (64.9%) were adult > 15 years of age and 64.1% were male (Table 1).

**Table 1:** Sociodemographic characteristic of the study participants (*n* = 245)

Variable	*N*	%
Age (in years)		
Median (IQR)	20 (13.5–28)	
Mean (± SD)	21.6 (± 12.7)	
≤ 15	86	35.1
> 15	159	64.9
Sex		
Female	88	35.9
Male	157	64.0
Education		
≥ Secondary	77	31.4
Primary	91	37.2
No education	77	31.4
Work place		
Indoor	109	44.5
Outdoor	136	55.5
Duration of stay in the area		
≤ 3 year	140	57.1
> 3 years	105	42.9
Season		
Dry	75	30.6
Wet	170	69.4

**Fig. 1:**
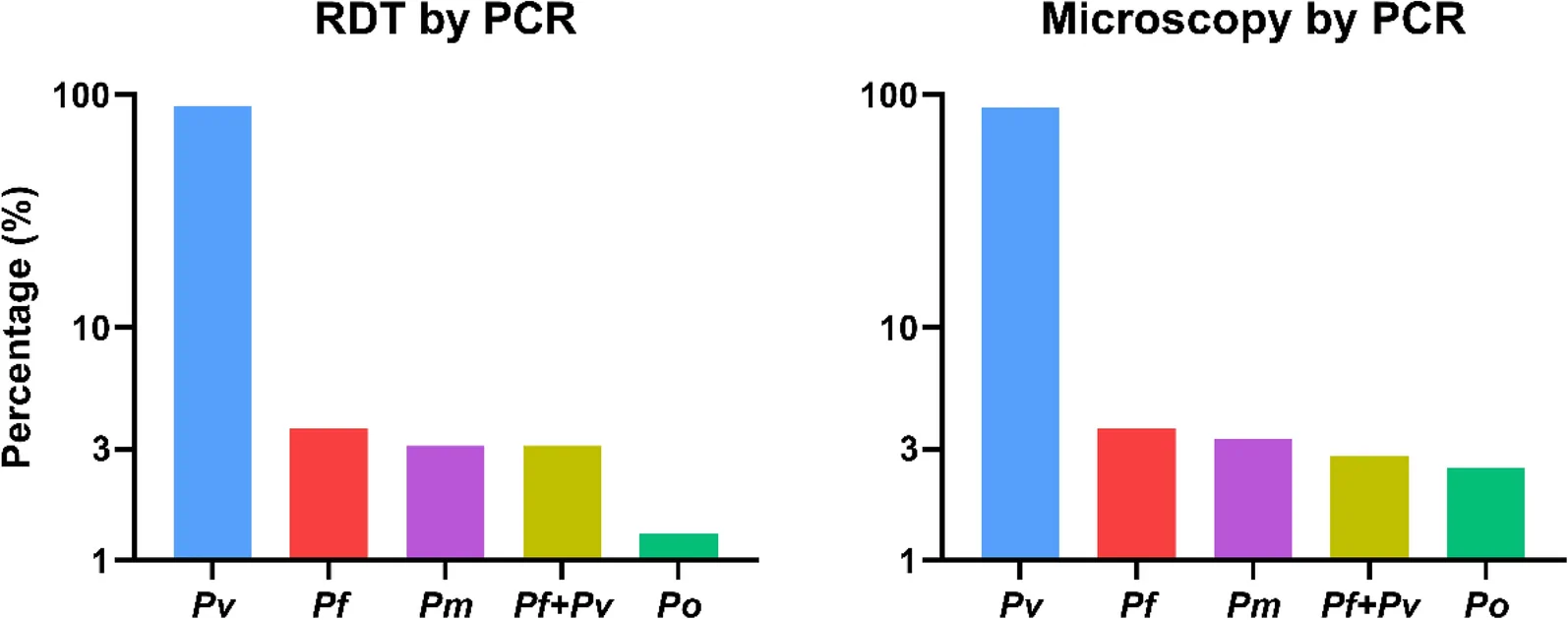
Percentage (logarithmic vertical scales) of malaria cases reported as Pv by microscopy (*n* = 245) and RDT (*n* = 162) confirmed by qPCR as Pv, Pf, Pm, mixed infection Pf + Pv and Po in Arjo-Didessa sugar development area, southwestern Ethiopia

All 245 samples were confirmed to be *Plasmodium* positive by qPCR. Among these 245 qPCR-confirmed cases, 215 (87.7%) were positive for Pv, 9 (3.7%) for Pf, 8 (3.3%) for Pm, 6 (2.5%) were positive for Po*,* and 7 (2.8%) had mixed infections (Pf and Pv) (Fig. 1). All Pm and Po infections were detected by qPCR were initially reported as Pv by microscopic examination or RDT. When the samples were stratified by patient age, the majority of Pm and Po infections were in adult > 15 years of age (11/159, 6.9% infection rate). However, males (13/157, 8.3%) and those working outdoors (12/136, 8.8%) were affected. In addition, infection was (8/75, 10.7%) in dry season (Table 2).

**Table 2:** Sex and age specific *Plasmodium* prevalence among 245 study participants in Arjo-Didessa sugar development area, southwestern Ethiopia, 2019–2021

Variable	Total enrolled	*Pv*, *n* (%)	*Pf*, *n* (%)	*Pm*, *n* (%)	*Pf* + *Pv*, *n* (%)	*Po*, *n* (%)
Sex						
Female	88	79 (89.8)	3 (3.4)	1 (1.1)	5 (5.7)	0
Male	157	136 (86.6)	6 (3.8)	7 (4.5)	2 (1.3)	6 (3.8)
Age group (in year)						
≤ 15	86	78 (90.7)	2 (2.3)	1 (1.2)	3 (3.5)	2 (2.3)
> 15	159	137 (86.2)	7 (4.4)	7 (4.4)	4 (2.5)	4 (2.5)
Work place						
Indoor	109	103 (94.5)	2 (1.8)	0	2 (1.8)	2 (1.8)
Outdoor	136	112 (82.4)	7 (5.2)	8 (5.9)	5 (3.7)	4 (2.9)
Season						
Dry	75	59 (78.7)	6 (8.0)	4 (5.3)	2 (2.7)	4 (5.3)
Wet	170	156 (91.8)	3 (1.8)	4 (2.4)	5 (2.9)	2 (1.2)

### Comparison among diagnostic methods

From the 245 vivax malaria cases, RDT was performed for 162 sub-samples, among them 8.6% (14/162) were negative by RDT (Supplementary Table S2). From the RDT positive cases qPCR was performed and confirmed 144 (88.8%, 95% CI 83.01–93.28) positive for Pv, 6 (3.7%, 95% CI 1.37–7.89) Pf, 5 (3.1%, 95% CI 1.01–7.06) Pm, 2 (1.2%, 95% CI 0.15–4.39) Po and 5 (3.1%, 95% CI 1.01–7.06) mixed infection (Pf and Pv) (Table 3). Moreover, all 245 microscopically examined cases were confirmed by qPCR. Of these 245 qPCR-confirmed cases, 215 (87.7%, 95% CI 82.98–91.58) were positive for Pv, 9 (3.7%, 95% CI 1.69–6.86) Pf, 8 (3.3%, 95% CI 1.42–6.33) Pm, 6 (2.4%, 95% CI 0.90–5.25) Po*,* and 7 (2.8%, 95% CI 1.16–5.80) mixed infection (Pf and Pv) (Table 3).

**Table 3:** Comparison of the performance of RDT, microscopy and qPCR for malaria diagnosis in Arjo-Didessa sugar development area, southwestern Ethiopia, 2019–2021

Diagnostic methods	qPCR					
	Pv, *n* (%)	Pf, *n* (%)	Pm, *n* (%)	Pf + Pv, *n* (%)	Po, *n* (%)	Total
RDT						
Negative	14 (100)	0	0	0	0	14
Pv	130 (87.8)	6 (4.1)	5 (3.4)	5 (3.4)	2 (1.4)	148
Total	144 (88.9)	6 (3.7)	5 (3.1)	5 (3.1)	2 (1.2)	162
Microscopy						
Pv	215 (87.7)	9 (3.7)	8 (3.3)	7 (2.9)	6 (2.5)	245
Total	215 (87.7)	9 (3.7)	8 (3.3)	7 (2.9)	6 (2.4)	245

## Discussion

Malaria elimination entails interruption of onward transmission by detecting every infection and undertaking proper case management [29]. Accurate parasite species identification is the cornerstone of proper treatment, case management, malaria intervention as well reduces the occurrence of drug resistance [26, 42]. In this study, among 245 qPCR-confirmed *Plasmodium*-positive cases, 3.3% Pm and 2.4% Po were detected*.* The present study reported the occurrence Pm for the first time in southwestern Ethiopia, while Po was previously reported by the same study team in 2019 community-based study as a submicroscopic infection [38]. Among the specimen positive for Pm and Po, 92.8% (13/14) were male, 78.6% (11/14) were adults above 15 years of age and 85.7% (12/14) were individuals who were engaged in outdoor activities. In addition, more than half (8/14) of these rare *Plasmodium* species occurred during the dry season.

The misidentification of Pm and Po by microscopy and RDT could lead to underestimation of the true burden of these parasites. Historically Pm and Po infections were presumed to be less than 1% in Ethiopia [30]. In this study, all Pm and Po infections were misidentified as Pv malaria by microscopy. In Ethiopia, microscopy is considered as the gold standard for malaria diagnosis. However, Pm and Po are commonly misdiagnosed by microscopy since microscopists are not often trained with rare *Plasmodium* species [30]. Different studies in Ethiopia also demonstrated misidentification of Pm and Po in the northwest [27], south [26], and southeastern Ethiopia [28]. This indicates that there is a need for new diagnostic tools with high specificity and accuracy to drive malaria elimination in low transmission settings like Ethiopia.

The finding of this study also revealed that higher prevalence of Pm and Po was recorded in adult male and outdoor workers. The result of this study was supported by a study from Papua Indonesia where Pm infection was higher among adults above 15 years of age [13]. However, studies in sub-Saharan Africa indicated that most of Pm and Po infection were documented in young children less than 15 years of age [3, 5, 18, 43–46]. In line with the present study, studies in Côte d’Ivoire and Mali reported dry season transmission of these malaria parasites [45, 46]. However, another study in Kenya showed no seasonal difference in the occurrence of both Pm and Po [47], while a study in Tanzania demonstrated transmission at the beginning of wet season [48]. More importantly, Pm is known to cause chronic, low-density infections that can persist for years, acting as a durable reservoir that can bridge dry seasons when *Anopheles* mosquito density is low. Po possesses hypnozoites, causing clinical relapses months or years after the infective bite, without new mosquito exposure. These biological features mean that transmission can be sustained sporadically and silently.

In this study, microscopic diagnosis misclassified 13 malaria cases (5.3%) as Pv mono-infections, while qPCR identified them as Pf mono-infections (7 cases) and Pf/Pv mixed infections (6 cases). In the same way, RDT misclassified 11 (6.8%) malaria cases as Pv mono-infections; however, the qPCR confirmed as Pf mono-infections (6 cases) and Pf/Pv mixed infections (5 cases). Consequently, those patients were inappropriately treated with chloroquine instead of the recommended artemisinin-based combination therapy (ACT) and single dose primaquine as per national guidelines. Such misclassification could potentially contribute to a reducing treatment efficacy and increased risk of severe malaria consequences, including mortality. The omission of a buffer in the preparation of Giemsa stain working solution across all laboratories in the study area could be a possible justification for misclassification of the parasites. Optimal staining of Giemsa can be attained by maintaining the pH of Giemsa, as it ensures clear visualization of parasite species-specific morphological features, such as Schüffner’s dots in Pv and Maurer’s clefts in Pf, which are essential for correct species identification. Similar findings linking inadequate staining to reduce microscopic sensitivity and species misidentification have been reported elsewhere in Ethiopia [26, 28]. In addition, misidentification by RDT could possibly be due to *hrp*2/3 genes deletions in the Pf species, which is reported in Ethiopia [49–51]. A key to an effective case management is an accurate species identification and proper treatment. Therefore, misidentification directly undermines treatment efficacy and can sustain transmission.

This study was not without limitation. This study used only Pv samples; however, negative samples, Pf positive, and mixed infection (Pf and Pv) were not included. If these samples were included, the prevalence might differ. The genetic diversity of Po was not done to identify *P. ovale wallikeri* and *P. ovale curtisi.*

## Conclusion

In conclusion, this study highlights the needs for a strategic shift to integrate Pm and Po species into Ethiopia's elimination program. First, in order to strengthen the diagnostic capacity, this includes a revised microscopy training modules by incorporating Pm and Po proper diagnosis, and the strategic deployment of a highly accurate diagnostic tool for all *Plasmodium* species that can be applied at the primary health care system. Secondly, all other *Plasmodium* species beyond the Pf and Pv must be identified and documented explicitly in the surveillance systems. Moreover, screening of outdoor laborers residing in irrigation development areas and sustained malaria surveillance both in high transmission season (wet season) and low transmission (dry season). Finally, treatment must ensure that any Pm or Po infection, once confirmed, should receive appropriate radical treatment in order to achieve sustainable malaria elimination in Ethiopia.

## Supplementary Information

Supplementary material 1: Table S1. Primer and probe sequences used in this study. Table S2. Comparison of the performance of RDTs and microscopy for malaria diagnosis.

## Data Availability

Data supporting the main conclusions of this study are included in the manuscript.
