# Supplementary material for: The hidden malaria: misidentification of Plasmodium malariae and Plasmodium ovale in low-transmission setting targeted for elimination in Ethiopia
Source: Parasit Vectors. 2026 Aug 3;19:464. doi: 10.1186/s13071-026-07511-0 (PMC13721312; doi:10.1186/s13071-026-07511-0)
Supplement: Supplementary file 1 [file 13071_2026_7511_MOESM1_ESM.docx]

# Supplementary

**Table S1**: Primer and probe sequences used in this study

| **Species-specific qPCRs (target: conserved regions in 18S rRNA)** | | |
| --- | --- | --- |
| **Species** | **Primer** | **Sequence (5’ -> 3’)** |
| *P. falciparum* | VarATS-F | CCCATACACAACCAAYTGGA |
|  | VarATS-R | TTCGCACATATCTCTATGTCTATCT |
|  | Pf-var-probe (FAM) | TRTTCCATAAATGGT |
| *P. falciparum* | Pf-1 | TATTGCTTTTGAGAGGTTTTGTTACTTTG |
|  | Pf-2 | ACCTCTGACATCTGAATACGAATGC |
|  | Pf-probe (FAM-BHQ) | ACGGGTAGTCATGATTGAGTT |
| *P. vivax* | Pv-1 | CGCTTCTAGCTTAATCCACATAACTG |
|  | Pv-2 | AATTTACTCAAAGTAACAAGGACTTCCAAG |
|  | Pv-probe (VIC-MGB) | CGCATTTTGCTATTATGT |
| *P. ovale* | Po-1 | AACCCAAAGACTTTGATTTCTCATAA |
|  | Po-2 | CCGACTAGGTTTTGGATGAAAGATTTTT |
|  | po-probe (NED-MGB) | CGAAAGGAATTTTCTTATT |
| *P. malariae* | Pm-1 | AGTTAAGGGAGTGAAGACGATCAGA |
|  | Pm-2 | CAACCCAAAGACTTTGATTTCTCATAA |
|  | Pm-probe (VIC-MGB) | ATGAGTGTTTCTTTTAGATAGC |

**Table S2.** Comparison of the performance of RDTs and microscopy for malaria diagnosis in Arjo-Didessa sugar development area, southwestern Ethiopia, 2019-2021

| **Methods** | | **Microscopy** | | | |
| --- | --- | --- | --- | --- | --- |
|  |  | ***Pv*, n(%)** | ***Pf*, n(%)** | ***Pf+Pv*, n(%)** | **Total** |
|  | **Negative** | 14 (100) | 0 | 0 | 14 |
| **RDTs** | ***Pv*** | 148 (100) | 0 | 0 | 148 |
|  | **Total** | 162(100) | 0 | 0 | 162 |
